# Holistic understanding of contemporary ecosystems requires integration of data on domesticated, captive and cultivated organisms

**DOI:** 10.3897/BDJ.9.e65371

**Published:** 2021-06-15

**Authors:** Quentin Groom, Tim Adriaens, Sandro Bertolino, Kendra Phelps, Jorrit H Poelen, DeeAnn Marie Reeder, David M Richardson, Nancy B Simmons, Nathan Upham

**Affiliations:** 1 Meise Botanic Garden, Meise, Belgium Meise Botanic Garden Meise Belgium; 2 Centre for Invasion Biology, Department of Botany and Zoology, Stellenbosch University, Stellenbosch, South Africa Centre for Invasion Biology, Department of Botany and Zoology, Stellenbosch University Stellenbosch South Africa; 3 Research Inst. for Nature and Forest (INBO), Brussels, Belgium Research Inst. for Nature and Forest (INBO) Brussels Belgium; 4 Department of Life Sciences and Systems Biology, University of Turin, Torino, Italy Department of Life Sciences and Systems Biology, University of Turin Torino Italy; 5 EcoHealth Alliance, New York, United States of America EcoHealth Alliance New York United States of America; 6 Ronin Institute for Independent Scholarship, Montclair, United States of America Ronin Institute for Independent Scholarship Montclair United States of America; 7 Bucknell University, Lewisburg, United States of America Bucknell University Lewisburg United States of America; 8 Department of Mammalogy, Division of Vertebrate Zoology, American Museum of Natural History, New York, United States of America Department of Mammalogy, Division of Vertebrate Zoology, American Museum of Natural History New York United States of America; 9 Arizona State University, Tempe, United States of America Arizona State University Tempe United States of America

**Keywords:** Darwin core, interoperability, invasive species, One Health, urban ecology

## Abstract

Domestic and captive animals and cultivated plants should be recognised as integral components in contemporary ecosystems. They interact with wild organisms through such mechanisms as hybridization, predation, herbivory, competition and disease transmission and, in many cases, define ecosystem properties. Nevertheless, it is widespread practice for data on domestic, captive and cultivated organisms to be excluded from biodiversity repositories, such as natural history collections. Furthermore, there is a lack of integration of data collected about biodiversity in disciplines, such as agriculture, veterinary science, epidemiology and invasion science. Discipline-specific data are often intentionally excluded from integrative databases in order to maintain the “purity” of data on natural processes. Rather than being beneficial, we argue that this practise of data exclusivity greatly limits the utility of discipline-specific data for applications ranging from agricultural pest management to invasion biology, infectious disease prevention and community ecology. This problem can be resolved by data providers using standards to indicate whether the observed organism is of wild or domestic origin and by integrating their data with other biodiversity data (e.g. in the Global Biodiversity Information Facility). Doing so will enable efforts to integrate the full panorama of biodiversity knowledge across related disciplines to tackle pressing societal questions.

## Introduction

Even by conservative estimates, 29% of the global land surface has been significantly modified by anthropogenic activities (Ellis 2011). On some continents, such as Europe, the percentage is much higher (Ellis et al. 2020). Agriculture, urbanisation and forestry are all anthropogenic activities that create or radically transform ecosystems. Furthermore, people further modify these ecosystems through the introduction and management of animals and plants. Domesticated animals, captive animals and cultivated plants are introduced for the production of food and other materials, but also for other qualities, including companionship, beauty, entertainment and shelter. Even semi-natural ecosystems are often maintained by domestic herbivores to restore ecosystem function and conserve habitats. Vast areas are grazed by cattle and sheep, while others are planted with food crops, such as wheat, corn, soybean, sunflower, sugar cane and rice or commercial forests of (mostly non-native) trees for timber and other forest products. Moreover, urban ecosystems are where a wide array of plant species, many of which are non-native, are cultivated in gardens for their aesthetic qualities. Such plants create important habitats and food sources for insect pollinators and other animals. Most of the earth’s human population lives in what is effectively an anthropogenic biome in which introduced organisms constitute a high proportion of the total biomass. For example, the biomass of livestock on the planet has been calculated to be more than an order of magnitude larger than the biomass of all wild mammals (Bar-On et al. 2018).

In this context, we examine the importance of integrating data on domestic and captive animals and cultivated plants by reviewing interactions with their wild counterparts. We also demonstrate how some citizen science projects reject or actively discourage observations of domestic, captive and cultivated organisms and how biodiversity data, collected by agriculture, horticulture and veterinary disciplines, are not integrated with other biodiversity datasets.

Here we briefly review the importance of data on the distributions and populations of domestic organisms for tackling some of the global ecological challenges and we make recommendations as to how the situation can be improved. We define domestic organisms as those organisms that would not exist at a particular location were it not for human intervention and where every part of their life cycle is managed, including their food, shelter, reproduction and ultimately harvesting, by humans. Despite the intense management of domestic organisms, interactions with wild organisms frequently occur and consequently play an integral role in shaping ecosystems.

## Predation, parasitism and herbivory

Domestic organisms can have significant negative impacts on native biodiversity when they are allowed to roam freely. In Italy, as in many countries, domestic cats (*Felis catus*) predate more than 200 other species, routinely killing birds, mammals, reptiles and amphibians (Mori et al. 2019). Domestic dogs (*Canis lupus familiaris*) can be an equally important predator (Holderness-Roddam and McQuillan 2014) and cause major disturbance to wildlife (Banks and Bryant 2007, Weston and Stankowich 2013). Domestic animals can also be the target of predation and parasitism from wild animals (Gazzola et al. 2005, Walker et al. 2018). Agricultural ecosystems can “subsidize” predators, which then return to adjacent wild ecosystems and impact wild prey species (Rand et al. 2006). For example, in the case of vampire bats (*Desmodus rotundus*) in Argentina, their population is twice as large in cattle-producing ecosystems compared to natural ecosystems, presumably due to the high density of an additional source of food (Delpietro et al. 1992). Furthermore, subsidies of food from domestic livestock can shift the diet of apex predators away from wild prey and, as a consequence, wild prey populations are no longer controlled by predators (Ciucci et al. 2020).

Herbivory by livestock can also have a major impact on ecosystems. Grassland covers between 12% and 21% of the global land surface and the population of cattle is close to a billion head (FAO 2020, Robinson et al. 2011). Expanding livestock production necessitates the conversion of existing ecosystems, such as slash-and-burn methods used to clear forests, replacing native grasslands with non-native pasture plants or introducing livestock in natural grasslands to create additional pasture for cattle grazing. Anthropogenic ecosystems are often a complex patchwork of land-use types, often with distinct boundaries between the different management regimes, including grazing (Hobbs et al. 2014). Nevertheless, spillover of herbivores between natural and anthropogenic ecosystems is extensive and goes in both directions (Blitzer et al. 2012).

The direct impacts of domestic organisms on ecosystems do not just involve mammals. Fish and shellfish are frequently stocked in natural waterways and coastal areas for recreational fishing, biocontrol or their aesthetic qualities. Introduced fish can alter natural ecosystems through interactions with native species, including increased competition and/or predation. For example, stocked brown trout (*Salmo trutta*) can reduce native invertebrate communities, even if those stocked fish are unable to create viable populations (Alexiades and Kraft 2017). Cultivated crops and other non-native trees and garden plants are a significant component of many anthropogenic ecosystems. They provide critical food resources for many wild species, particularly where habitat has been reduced through fragmentation (e.g. Chaves and Bicca-Marques 2017). Crop pests are also an important source of food for many animals, such as Brazilian free-tailed bats (*Tadarida brasiliensis*) which feed extensively on corn earworm moths (*Helicoverpa zea*) (McCracken et al. 2012).

The characteristics of cultivated plants and the way that they are grown is likely to have a large influence on whether the plants have a positive or negative impact on wild organisms. For example, crop and forestry monocultures can have negative consequences for wild bees, whereas domestic gardens may provide benefits (Kaluza et al. 2016, Samnegård et al. 2011). Furthermore, the keeping of domesticated bees results in direct competition with native pollinators (Ropars et al. 2019). Plant cultivation can indirectly affect vertebrates by changing the abundance and species composition of their arthropod prey. For example, the reduced breeding success of the insectivorous Eurasian blue tit (*Cyanistes caeruleus*) in urban ecosystems in comparison to congeners in native woodland has been attributed to the reduced population densities and lower diversity of arthropods on non-native cultivated trees (Helden et al. 2012, Pollock et al. 2017).

## Genetic impacts

Hybridization between wild organisms and their domestic counterparts is widely perceived as a threat to the conservation of native biodiversity. It occurs, for example, between wild canids and domesticated dogs (Leonard et al. 2013) and between wild and domestic mink (*Neovison vison*) (Kidd et al. 2009) and, in both examples, the introgressed alleles may be deleterious for threatened wild populations. Similarly, stocking and aquaculture of fish can have a negative effect on the genetic diversity of wild populations of those species (Bourret et al. 2011, Bolstad et al. 2017, Gossieaux et al. 2019). Hybridization is also an issue for gene flow between crops and their wild relatives, such as potatoes (*Solanum* sp.) in the Andes (Scurrah et al. 2008). In agroecosystems, it has been suggested that the traits selected during the domestication of crop plants can disrupt species interactions and can create selective pressures that can drive the evolution of wild organisms (Macfadyen and Bohan 2010). Hybridization is widely acknowledged as “a stimulus for the evolution of invasiveness in plants” (Ellstrand and Schierenbeck 2000).

In contrast, others see the hybridization of closely related wild and domestic species brought into “artificial sympatry” not as a threat to genetic integrity, but as a mechanism whereby new biological entities are created that could, conceivably, be better suited than native species to new, human-dominated environments (Thomas 2013). Regardless of the directionality of genetic influences of domestic-wild hybridization, collection of data on the domestic organisms in question and on the interactions of domestic and wild organisms is critically important.

## Wildlife disease

There is ample evidence for the interchange of infectious diseases between domestic animals, including livestock (Frölich et al. 2002, Martin et al. 2011) and pets, such as cats and dogs (Clark et al. 2018, Wells et al. 2018), wild animals and humans. As an example, domestic dogs are a reservoir for Guinea worm (McDonald et al. 2020), Rickettsial diseases (Levin et al. 2012, Ng-Nguyen et al. 2020), Leishmaniases (Grimaldi and Tesh 1993), rabies virus (Lembo et al. 2008), Chagas disease (*Trypanosoma cruzi*) (Gürtler et al. 2007), *Strongyloides stercoralis* (Sanpool et al. 2020) and others. Likewise, domestic cats can transmit more than 20 diseases to humans and wild animals (Lepczyk et al. 2015). Many of these diseases are zoonotic and can cause serious illness and/or mortality in human populations. There are other examples from livestock, such as domestic pigs (*Sus scrofa domesticus*) mediating the transmission of the deadly Nipah virus (*Nipah henipavirus*) from fruit bats (*Pteropus* spp.) to farmers (Pulliam et al. 2012). Indeed, domestic mammals hold a central place in the network of known mammal virus associations (Wells et al. 2018). In the case of domestic chicken flocks, there is ample evidence for the exchange of viral diseases in both directions with wild birds (e.g. avian influenza) (Scott et al. 2018, Ferreira et al. 2019).

In aquatic ecosystems, aquaculture facilities not isolated from wild ecosystems have the potential to increase disease in wild fish populations. This might occur through disease spillover to wild congeners of farmed species or to other species. Captive fish populations can act as reservoirs of disease or otherwise affect disease dynamics in nearby wild populations (Bouwmeester et al. 2020). Similarly, the introduction of domesticated bees can transmit disease to wild bee species, and can even lead to local extinction of some wild species (Graystock et al. 2016, Meeus et al. 2018). Even cultivated plants can act as reservoirs of pests and diseases to wild plants, such as the spread of Knopper gall wasps (*Andricus quercuscalicis*) infesting English oak trees (*Quercus robur*) in northern Europe which is mediated through the planting of its alternate host Turkey oak (*Q. cerris*) (Hails and Crawley 1991).

## Potentially invasive species

Cultivated plants, pets, wildfowl collections and aquarist collections are among the largest sources of invasive species (Funnell et al. 2009, Lockwood et al. 2019, Niemiera and Holle 2009). Urban ecosystems are foci for introductions of non-native species and frequently act as launching sites for invasions into surrounding natural ecosystems (Alston and Richardson 2006, McLean et al. 2017). Knowledge of species that are kept domestically or cultivated is useful for calculating the potential risk of escape and the possibility of a species becoming invasive. Arboreta and other collections of non-native species, typically located in urban ecosystems, provide opportunities to serve as sentinel sites for the identification of incipient invasions (e.g. Fanal et al. 2021). However, few databases collate open information on organisms in homes, gardens, arboreta and other collections in any particular region. Sources, such as seed catalogues, pet shop surveys, border interception databases and import certificates, have been used to evaluate the propagule pressure of potentially invasive species (Liang et al. 2006, Kopecký et al. 2013, van Kleunen et al. 2018). However, these sources of data tell us little about the lifespan, fecundity and frequency of pets and garden plants. As a consequence, horizon scans and risk assessments rely on scant information on trade in these organisms, but have virtually no information on the size and geographic distribution of captive populations (Bertolino et al. 2020). If observations of non-native garden plants were available, they would inform us of the environmental tolerances of these species, their co-occurrence and their interactions with other native and non-native organisms. Furthermore, ecological and economic impacts of invasive species are highly correlated across taxa and regions (Vilà et al. 2010). Therefore, data on domesticated and cultivated organisms are important for impact studies. As an example, the Asian hornet (*Vespa velutina*) has negative impacts on apiculture through predation at beehives (Monceau et al. 2014), yet data on the presence of the approximately 90 million global beehives of *Apis* (FAO 2020), often set out in natural environments or gardens, are not readily available.

## Urban ecology and agroecology

Urban ecosystems and gardens are unique and interesting in their own right (Adler and Tanner 2013). In these habitats, cultivated plants and captive animals co-exist and interact directly with wild biodiversity, both native and non-native. Domestic gardens are the one type of ecosystem that most people manage; as such, their management decisions have a direct influence on local biodiversity, including the species they cultivate, the pets they keep, the birds they feed, the nest boxes and insect hotels they erect and the garden products they use (Sandström et al. 2006, Yang et al. 2019). In some highly urbanised areas, such as Flanders in Belgium, gardens occupy more total land surface than areas under conservation management, like nature reserves and forests (Vught et al. 2020). Urban ecosystems are increasingly seen as making an important contribution to climate change adaptation, ecosystem services and food security (Aerts et al. 2016, Eigenbrod and Gruda 2015). Likewise, biodiversity in managed agricultural ecosystems contributes to ecosystem services, such as pollination, soil nutrient cycling, watershed protection and carbon sequestration and many people come into contact with biodiversity in and around farmland (Jarvis et al. 2007). Schlaepfer et al. (2020) emphasise the importance of non-native trees for their intrinsic value and their contributions to human well-being. In contrast, Potgieter et al. (2019) highlight how non-native woody plants contribute to changes in vegetation structure, sometimes even enhancing criminal activity in urban areas. Urban agroecology, the study of urban food systems, links both realms and is expected to quickly grow as a valued discipline (Altieri and Nicholls 2018). The study and management of biological invasions in urban areas require insights into the full spectrum of biodiversity that occurs in these regions (Gaertner et al. 2017).

**Figure 1.**
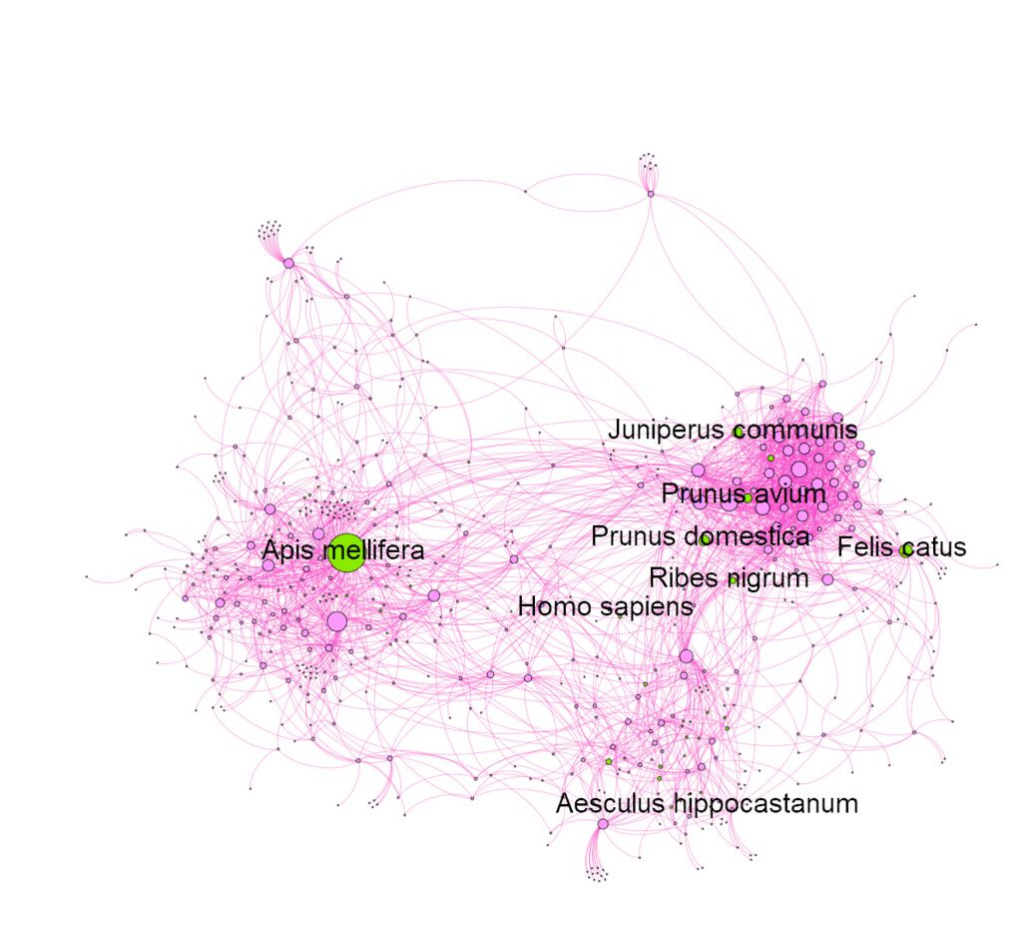
A species interaction network of the organisms recorded for Meise Botanic Garden in Belgium. It demonstrates how the people, cultivated plants and domesticated animals (green nodes) are integrated into the ecosystem of the Garden through their interactions with wild organisms (pink nodes). Species included are only those available on GBIF (GBIF 2021) that have been recorded in the Garden and their potential interactions are those available in GloBI (Poelen 2021, Poelen et al. 2014). Nodes are proportional to the network degree of the organism's interactions and the eight domesticated or planted species are labelled by name. The code used to generate this network is available (Groom 2021).

As a demonstration of the importance of domestic organisms in urban ecosystems, we constructed a species interaction network for wild and cultivated organisms recorded at Meise Botanic Garden in Belgium. Only two domesticated animals are present in the Garden, honey bees (*Apis mellifera*) and domestic cats from neighbouring houses (Fig. 1). This network demonstrates that these two species have among the largest number of potential interactions with other organisms in the garden. Indeed, honey bees have the highest "betweenness centrality" of any species in the network. Betweenness centrality is a measure of how central a vertex is in a network, based on the number of shortest paths that travel through it. While this is only a network of potential interactions, the possibility for real impacts on the wild organisms of the Meise Botanic Garden is large.

## Observations of domestic and cultivated species

Volunteers are a major contributor to ecological and biogeographic data (Chandler et al. 2017, Irwin 2018, Poisson et al. 2020). The internet and smartphones have dramatically increased the possibilities for public involvement in research and so have the types of projects and types of data gathered (Adriaens 2015, Theobald et al. 2015). For some taxa, such as birds, these internet resources have become the primary source of ecological and distributional data (Sullivan et al. 2009). Given the overwhelming evidence that domestic animals and cultivated plants are an integral part of global ecosystems and that they are often the dominant species, why is it that we actively discriminate against domesticated organisms when collecting data on biodiversity? Most recording platforms targeting the naturalist community primarily aim to record only observations of wild organisms and actively reject data on domestic or cultivated species. For example, the international biodiversity recording platform, iNaturalist, states:

“The main reason we try to mark things like this [captive/cultivated] is because iNat is primarily about observing wild organisms, not animals in zoos, garden plants, specimens in drawers, etc., and our scientific data partners are often not interested in (or downright alarmed by) observations of captive or cultivated organisms. ”

Any observation on iNaturalist marked captive/cultivated will never reach “Research Grade”. It will, therefore, not be transferred to GBIF, even if the species identification is validated. It is germane that iNaturalist puts the responsibility for this decision on their “scientific data partners”. They are not alone – eBird, the single largest contributor to GBIF, explicitly requests users not to record captive birds, escaped pets, domestic fowl and pet birds (Sullivan et al. 2009). These platform policies to include only wild organisms are not exceptional. There is considerable controversy over what should be recorded (and where), leading some local citizen science organisations to write clarifying guidelines (Walker et al. 2015).

Other citizen science initiatives have bucked the trend and have specifically tried to survey the occurrence of alien and native plants in gardens (e.g. Dehnen-Schmutz and Conroy 2018, Pergl et al. 2016). Such surveys provide a measure of propagule pressure or the potential of introduced species to establish and thrive, which may explain the establishment success of these species outside gardens.

The gaps in available data on domestic/captive/invasive species are plainly evident in GBIF. For example, there are approximately 26 billion chickens (*Gallus gallus domesticus*) in the world (FAO 2020), but only 55,000 observations on GBIF. For comparison, the rare, endangered and localised bearded vulture (*Gypaetus barbatus*) has almost the same number (54,000). Clearly, recording chickens in commercial chicken barns may not be useful for ecological analyses, but recording free-ranging chickens in rural and urban ecosystems may well be.

## The causes and solutions

There is no doubt that all organisms, be they native, non-native, growing wild, in captivity or in cultivation, are important components of biodiversity. Suggestions on how to deal with data in these different categories have generated lively debate among biologists. For example, Schlaepfer (2018), in a paper entitled “Do non-native species contribute to biodiversity?” proposed that “biodiversity and sustainability indices should include all species”. This suggestion was vigorously opposed by a group of invasion ecologists who argued that this approach “will reduce our capacity to detect the effects of non-native species on native biodiversity with potentially devastating consequences” (Pauchard et al. 2018). There are many other examples of vigorous debate in literature on the hazards and opportunities implicit in mixing such data for various purposes (Feest et al. 2010). Schlaepfer (2018) does not clarify whether he includes domestic organisms in his view of biodiversity, but many of his arguments still apply.

Part of the reason for the artificial demarcation between wild and domestic/cultivated organisms is the divisions of research domains, industrial sectors and their respective regulatory bodies. Researchers and managers in agriculture, animal husbandry, the pet trade, epidemiology, conservation, forestry, ecology and invasion science are all interested in these data, but also generate data for their own needs. Traditionally, biodiversity observation data have been the preserve of biogeographers and conservationists and observations of cultivated and domesticated organisms are removed before creating maps and building distribution models (Gueta and Carmel 2016). Yet, as the examples above show, these data have much broader uses in research than just biogeography and conservation. Indeed, one cannot hope to understand and predict the dynamics of contemporary ecosystems without also considering the domesticated, captive and cultivated components of “the whole landscape” (sensu Hobbs et al. 2014).

For at least the past 400 years, Western culture has considered the realms of humans and nature as separate (Paterson 2006). Indeed, it has been suggested that mobile biodiversity recording apps reinforce this artificial division between humans and nature by neglecting the human-influenced aspects of nature (Altrudi 2021). Nevertheless, in recent years, the One Health approach has emerged to bring together different sectors to work together to improve human and animal health in the context of a shared environment (Atlas 2012). This approach applied to biodiversity observations would see a marked improvement in reducing the barriers that prevent the full integration of data. One could even extend this concept under a banner of ‘One Biodiversity’ given that the same principles of an interconnected whole apply.

Another reason for observations of domesticated organisms being excluded from biodiversity datasets is that there has lacked a means by which these observations can be distinguished from those of wild organisms. The preeminent standard used to communicate biodiversity observations is the Darwin Core standard (Wieczorek et al. 2012). Until recently, there were no unambiguous or standardised methods in Darwin Core to indicate that the organism observed was captive or cultivated; however, this oversight has now been changed: The Biodiversity Information Standards organisation recently ratified a proposal to add the term "degreeOfEstablishment" to the standard and for this term to use a vocabulary adapted from Blackburn et al. (2011) (see Groom et al. 2019). The publishing tools and data infrastructure, run by GBIF, will be adapted to support these new Darwin Core terms during 2021.

It is unreasonable to expect systematic observation of all domesticated organisms to be collected. Indeed, projects devoted to the study of wild organisms do not want to be swamped with large numbers of observations of pets and garden plants. However, some of these data are already collected by national and regional authorities for veterinary and agricultural statistics, pathogen surveillance and animal welfare (Table 1). Yet these data are poorly integrated with biodiversity data and are often inaccessible to biodiversity researchers. Recognition by the relevant authorities that these are important data for ecologists would help drive access to these data. Great adherence to the FAIR data principles of being Findable, Accessible, Interoperable and Reusable would improve the situation (Wilkinson et al. 2016). This would mean greater use of community standards, stable identifiers and particularly full description of the data with metadata.

Given that a data standard now exists (i.e. Groom et al. 2019), we now recommend that data collectors and providers do not reject any data based on the organism’s status of cultivation, captivity or domestication, but rather ensure that its status is adequately described using Darwin Core. Furthermore, we recommend the greater integration of all data on biodiversity, whether it is of wild or domestic origin. These data may include information on the species kept as pets, farm animals, garden plants and crops, but also pests and diseases of those species. Indeed, there is clearly much to be gained from encouraging the collection and sharing of such data on domestic organisms, their distributions, abundance, behaviour and interactions with wildlife.

In conclusion, although it is fairly self-evident to an ecologist that domestic organisms are part of ecosystems, data on these organisms remain poorly integrated into global data systems and are thus often disregarded. Yet, these data are highly relevant to solving many environmental challenges and should, therefore, be more actively gathered and shared.

## Figures and Tables

**Table 1.** Examples of datasets related to domestic organisms that could be incorporated into biodiversity datasets if correctly documented and standardised.

**Sector**	**Type**	**Example**
Agriculture	Crop map	https://data.ca.gov/dataset/statewide-crop-mapping https://data.linz.govt.nz/layer/50307-nz-orchard-polygons-topo-150k/
	Livestock survey	https://data.gov.jm/dataset/farmer-reports/resource/bcc809a3-92c7-411e-bb94-1d8571c55f78 https://data.gov.sa/Data/en/dataset/estimated-number-of-goats--by-administrative-regions-2
	Aphid monitoring	https://www.sasa.gov.uk/wildlife-environment/aphid-monitoring
	Disease host specificity	https://www.apsnet.org/edcenter/resources/commonnames/Pages/default.aspx
Veterinary Science	Records of parasites, such as *Hypoderma* sp. (Warble fly) and *Fasciola hepatica* (liver fluke)	https://data.gov.uk/dataset/9607543e-2deb-41d7-ac9c-a38f952d31a7/other-species-conditions-data
	Bees hive inspections for parasites	https://data.defra.gov.uk/Agriculture/APHA0365-Apiary_Inspections_for_Exotic_Pests_2012.csv
Horticulture	Inventory of botanic garden	https://www.bgci.org/resources/bgci-databases/plantsearch/
	Observations of garden plants	http://doi.org/10.5281/zenodo.3514685
Domestic animals	Pets census	https://datos.gob.es/en/catalogo/l01082798-animales-de-compania1
	Zoo inventory	http://cza.nic.in/page/en/inventory-of-animals-in-zoos https://www.rzss.org.uk/downloads/agm/2013/EZ_Inventory_AGM2013.pdf Species360 Zoological Information Management System (ZIMS) (zims.Species360.org) www.zootierliste.de
